# Early and Sensitive Detection of Pathogens for Public Health and Biosafety: An Example of Surveillance and Genotyping of SARS-CoV-2 in Sewage Water by Cas12a-Facilitated Portable Plasmonic Biosensor

**DOI:** 10.34133/research.0205

**Published:** 2023-07-28

**Authors:** Tianzhong Li, Yuzhi Chen, Zhi Chen, Yuan Hao, Minyi Liang, Yingxia Liu, Guanyong Ou, Huanian Zhang, Yuxuan Tang, Yabing Hao, Swelm Wageh, Omar A. Al-Hartomy, Abul Kalam, Bin Zhang, Xin Shi, Xuejin Li, Han Zhang

**Affiliations:** ^1^ College of Physics and Optoelectronic Engineering, Institute of Translational Medicine, Department of Otolaryngology, Shenzhen Second People’s Hospital, First Affiliated Hospital of Shenzhen University, Health Science Center, Shenzhen University, Shenzhen 518060, China.; ^2^ Shenzhen Key Laboratory of Sensor Technology, Shenzhen 518060, China.; ^3^ The Sixth Affiliated Hospital of Guangzhou Medical University, Qingyuan People's Hospital, Qingyuan 511518, China.; ^4^ Shenzhen Key Laboratory of Pathogen and Immunity, National Clinical Research Center for Infectious Disease, State Key Discipline of Infectious Disease, Shenzhen Third People's Hospital, Second Hospital Affiliated to Southern University of Science and Technology, Shenzhen 518112, China.; ^5^ School of Medicine, Southern University of Science and Technology, Shenzhen 518055, China.; ^6^ School of Physics and Optoelectronic Engineering, Shandong University of Technology, Zibo 255049, China.; ^7^ Department of Physics, Faculty of Science, King Abdulaziz University, Jeddah 21589, Saudi Arabia.; ^8^ Research Center for Advanced Materials Science (RCAMS), King Khalid University, P.O. Box 9004, Abha 61413, Saudi Arabia.; ^9^ Department of Chemistry, College of Science, King Khalid University, P.O. Box 9004, Abha 61413, Saudi Arabia.; ^10^ Health Sciences Institute, China Medical University, Shenyang 110000, China.; ^11^ The Chinese University of Hong Kong, Shenzhen 518060, China.

## Abstract

Infectious diseases severely threaten public health and global biosafety. In addition to transmission through the air, pathogenic microorganisms have also been detected in environmental liquid samples, such as sewage water. Conventional biochemical detection methodologies are time-consuming and cost-ineffective, and their detection limits hinder early diagnosis. In the present study, ultrafine plasmonic fiber probes with a diameter of 125 μm are fabricated for clustered regularly interspaced short palindromic repeats/CRISPR-associated protein (CRISPR/Cas)-12a-mediated sensing of severe acute respiratory syndrome coronavirus 2 (SARS-CoV-2). Single-stranded DNA exposed on the fiber surface is trans-cleaved by the Cas12a enzyme to release gold nanoparticles that are immobilized onto the fiber surface, causing a sharp reduction in the surface plasmon resonance (SPR) wavelength. The proposed fiber probe is virus-specific with the limit of detection of ~2,300 copies/ml, and genomic copy numbers can be reflected as shifts in wavelengths. A total of 21 sewage water samples have been examined, and the data obtained are consistent with those of quantitative polymerase chain reaction (qPCR). In addition, the Omicron variant and its mutation sites have been fast detected using S gene-specific Cas12a. This study provides an accurate and convenient approach for the real-time surveillance of microbial contamination in sewage water.

## Introduction

The emergence and spread of infectious diseases threaten biosafety worldwide. Since the start of the outbreak in late 2019, the coronavirus disease 2019 (COVID-19) pandemic has severely affected public health at a global scale [1]. The pathogenic agent of COVID-19, severe acute respiratory syndrome coronavirus 2 (SARS-CoV-2), is morphologically similar to the severe acute respiratory syndrome (SARS) and Middle East respiratory syndrome (MERS) pathogens. It shares 79.08% sequence similarity with SARS and >50% similarity with MERS [2]. Its genome consists of a single-stranded RNA (ssRNA) that encodes envelope protein (E), membrane protein (M), nucleocapsid (N), and spike protein (S) [3]. The nucleocapsid protein serves as a structural support for genome encapsulation, viral replication, and RNA transcription [4]. The spike protein mediates the recognition of angiotensin-converting enzyme 2 (ACE2) on host cells and facilitates the fusion between the virus and cell membrane [5]; it also induces the secretion of neutralizing antibodies [6], thus serving as a key target for vaccination and therapy. The rapid mutation frequency of S gene has resulted in a weakening of the host immune responses, thereby prolonging the pandemic [7]. Based on the World Health Organization (WHO) nomenclature system, 5 variants of concern (VOCs) have emerged in the pandemic: Alpha (B.1.1.7), Beta (B.1.351), Gamma (P.1), Delta (B.1.617.2), and Omicron (B.1.1.529). Compared with the other variants, Omicron exhibits a 70-fold greater propagation ability and a slower rate of replication [8]. Key mutations, including N501Y, D614G, H655Y, N679K, and P681H, have been annotated in its genome and serve as unique targets for viral detection [9]. Despite the weak respiratory symptoms caused by Omicron, its rapid diffusion and high infectivity demand worldwide surveillance and collaborative control.

Water contamination refers to the accumulation of hazardous substances in a water body that exceeds the capacity for self-clearance. Contaminants include household pollutants, pesticides, industrial wastes, antibiotics, and microorganisms, which not only cause economic loss but also threaten human health [10]. Both respiratory and gastrointestinal viruses can be detected in environmental water samples and serve as good indicators of pathogen transmission. For instance, the occurrence of SARS-CoV-2 in drainage system has already been confirmed [11]. However, the accurate sensing of extremely low numbers of genomic copies complicates early detection of viral particles in sewage water [12]. Quantitative polymerase chain reaction (qPCR) is considered as the gold standard among virus detection techniques because of its high sensitivity and specificity [13]. Either fluorescent dyes or probes can serve as indicators of viral existence. For nasopharyngeal swabs, a cycling threshold (Ct) higher than 35 is normally justified as a positive readout [14]. This method allows for fast detection of a large population, as several swabs can be mixed into one sample to reduce the processing time. Nevertheless, qPCR requires specialized personnel and facilities to obtain validated results and is sensitive to aerosol contamination. Other techniques, such as loop-mediated amplification (LAMP) [15], recombinase polymerase amplification (RPA) [16], and rolling circle amplification (RCA) [17], can generate sufficient DNA for detection, but with a high probability of false-positive results. To determine variant sublineages, high-throughput sequencing has delivered promising results when combined with multiplex reverse transcription PCR (RT-PCR) [18]. However, the undesirable detection limit and high cost hinder its application in large-scale variant classification. Electrochemical and optical diagnostic approaches have only been investigated in the laboratory because of the need for specialized equipment [19,20]. Considering the ongoing threat of COVID-19, novel detection techniques that are simple to operate, cost-effective, and less time-consuming are urgently needed.

Clustered regularly interspaced short palindromic repeats/CRISPR-associated protein (CRISPR/Cas) systems have been widely applied in biological and medical fields as gene editing tools [21,22]. CRISPR/Cas family members include Cas9, Cas12, and Cas13, which can recognize and cleave DNA/RNA with high specificity [23]. Cas9 assembles with guide RNA to facilitate double-stranded DNA (dsDNA) cutting and gene editing [24,25]. Unlike Cas9, Cas12 ribonucleoprotein (RNP) is composed of enzymes and CRISPR RNAs (crRNAs) and possesses only one RuvC domain [26]. In the Cas12-based one Hour Low cost Multipurpose highly Efficient System (HOLMES) assay, the identification of target dsDNA simultaneously activates the *trans*-cleavage of single-stranded DNA (ssDNA) reporters, which emit fluorescent signals for template dsDNA quantification [27]. Cas13 mainly cuts targeted RNA and *trans*-cleaves nonspecific ssRNAs to generate signals [28]. Gootenberg *et al.* [29] developed a specific high-sensitivity enzymatic reporter unlocking (SHERLOCK) assay based on Cas13 that produces fluorescent signals upon cleaving ssRNA to detect Zika and Dengue viruses. When combined with RT-PCR or isothermal amplification, Cas12/13 enzymes can be exploited to detect extremely low copy numbers of viruses [30]. Despite the fast-growing number of COVID-19 studies involving the CRISPR/Cas system, most of these investigations are fluorescence-based. To the best of our knowledge, methodologies of photonic CRISPR/Cas-based sensing (MoPCS), which combine the advantages of photonic devices and the specificity of the Cas enzyme, have outperformed traditional detection methods to date.

To accurately and sensitively read the highly specific results of CRISPR/Cas systems, a fast and portable microdetector is desirable. One promising candidate is the plasmonic fiber biosensor, which combines the advantages of both fiber-optic sensing and surface plasmon resonance (SPR) technology, including small size, rapid optical response, high sensitivity, label-free detection, and real-time surveillance [31]. In practice, it proves to be effective for immunodetection in the liquid phase. Liu *et al.* [32] recently designed a highly tilted fiber Bragg grating with gold fabrication for the specific recognition of environmental estrogens with a detection limit of 1.5 × 10^−3^ ng/ml. A transmission-type fiber-optic biosensor was previously developed to detect glucose in urine [33]. Apart from the Cas12-mediated fluorescent methods [34], no CRISPR and SPR combinatory sensing methodology exists for the detection of COVID-19 in water samples. To date, there are no reports of the sensing of viral DNA by CRISPR enzyme on plasmonic fiber probes.

**Fig. 1.**
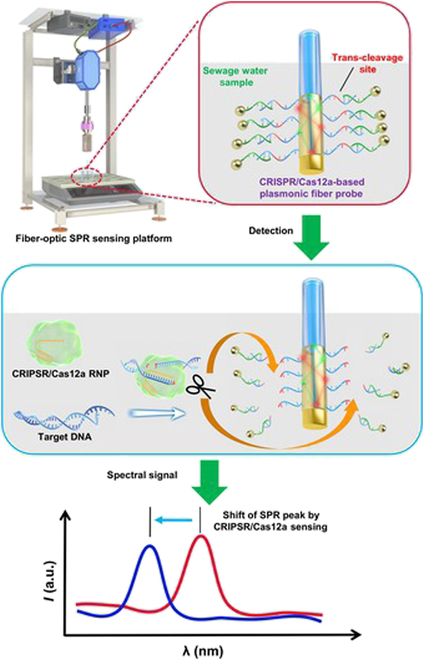
Schematic representation of the portable fiber-optic sensing platform for detecting SARS-CoV-2 in sewage water. The CRISPR/Cas12a-based plasmonic fiber probes are modified with AuNPs through DNA base complementarity. ssDNAs with *trans*-cleavage sites are exposed on the fiber probe. Target DNA activates Cas12a RNP and causes the *trans*-cleavage of ssDNA. The release of AuNPs from sensor surface leads to a shift of SPR peak. The corresponding spectral signals are transmitted in real time by the fiber probe.

In this study, a portable plasmonic fiber sensing device incorporating a CRISPR/Cas system was engineered for the real-time surveillance of SPR signals. Virus-containing sewage water samples from various sources were examined, and the relationship between viral copy number and SPR wavelength shift was analyzed (Fig. 1). Point mutations on the Omicron variant genome can be accurately recognized by the device under the guidance of site-specific Cas12a enzymatic complex. This study provides a miniaturized SPR system that promises accurate point-of-care testing (POCT) of SARS-CoV-2 in trace environmental samples, expediting the early control and prevention of infectious diseases.

## Results

### Assembly of the fiber-optic sensing platform

**Fig. 2.**
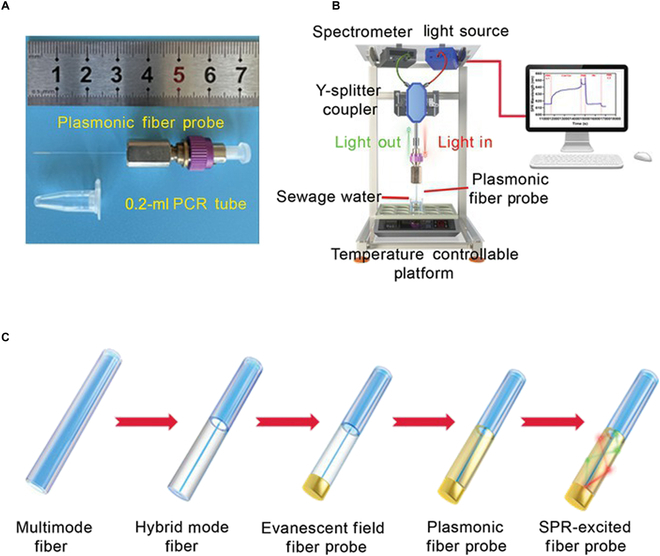
Assembly of the miniaturized SPR device and plasmonic fiber probe. (A) Structure of the 125-μm plasmonic fiber probe. (B) Front view of fiber-optic SPR sensing platform. (C) Engineering procedure of fiber probe.

A miniaturized plasmonic fiber biosensor was assembled to measure surface loading with ultrasensitivity [35]. A 125-μm fiber tip with a length of 5 mm was engineered to examine trace samples and allow for viral detection in a narrow space (Fig. 2A). The tip is excited by broadband white light in the visible range [36], which is reflected by an end-face mirror, and the output signal is received by a micro-spectrometer. A Y-splitter coupler was used to separate the incoming and outgoing light for accurate delivery of signals to the spectrometer. A thermostat was installed under a fiber probe to maintain the temperature for sensor fabrication and virus detection. During operation, SPR signals were collected in real time and displayed after spectral normalization (Fig. 2B). To assemble the plasmonic fiber probe, a multi-mode fiber (MMF) was connected to a single-mode fiber (SMF) to introduce a sufficient evanescent field on the SMF surface. An Au coating on the SMF portion provided both SPR excitation and versatility of surface fabrication (Fig. 2C).

**Fig. 3.**
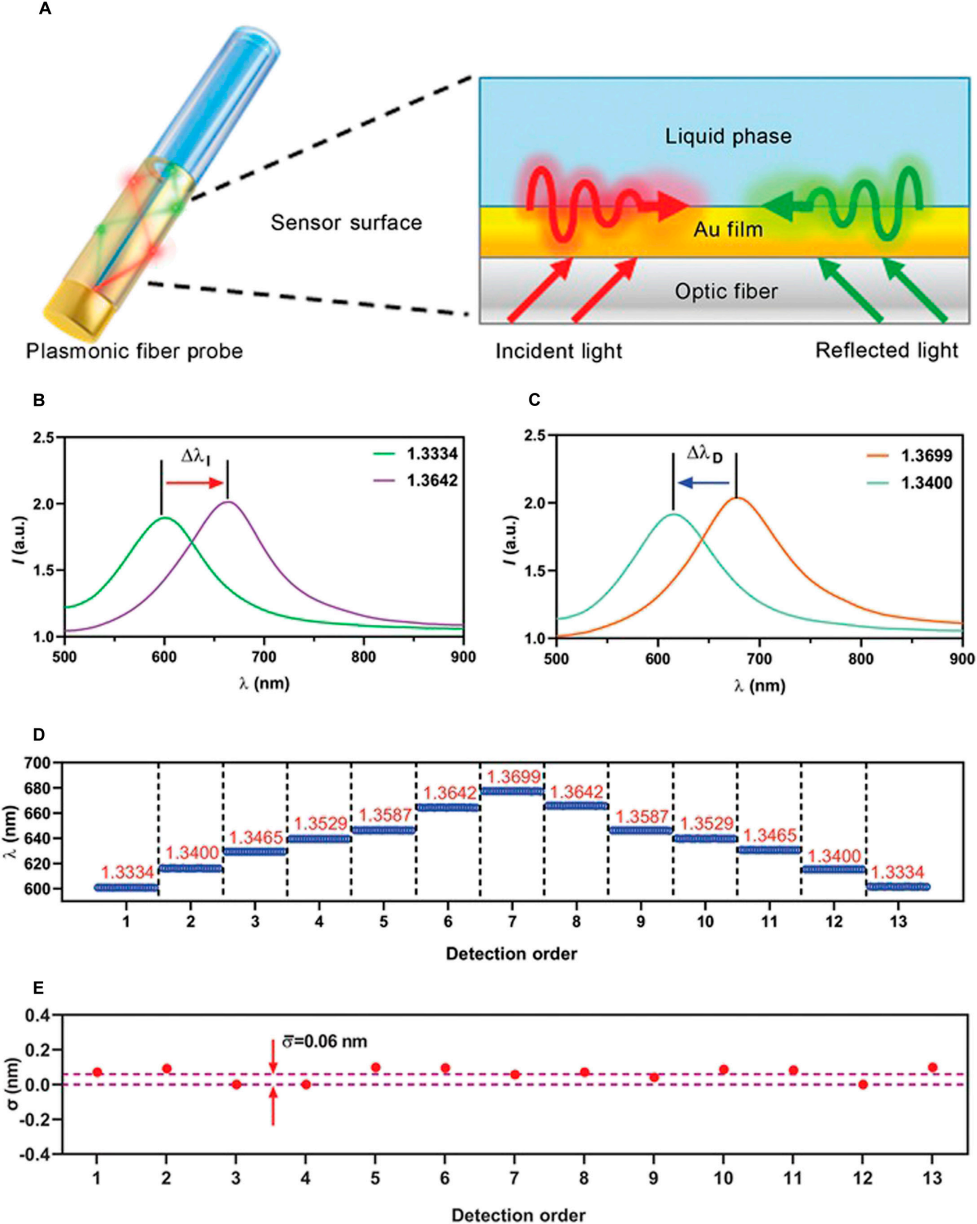
Sensing principle and sensor performance. (A) Graphical display of SPR excitation on the sensor surface. (B and C) SPR peak shifts due to the (B) increasing and (C) decreasing surface loading. (D) Reading of SPR peaks in liquid environment with different surface loadings. The refractive index of each liquid environment is marked in red. (E) Detection stability of fiber probe in different liquid phases. Detection time: 1 min.

A multi-/single-mode hybrid fiber delivered the transmitted light from MMF to SMF surface, stimulating SPR effects on the Au-coated SMF surface. As a result of the design of end-face mirror on the exit end of SMF, the optical signals were reflected on the SMF end, exciting the secondary SPR sensing. Subsequently, the reflected light carrying SPR signals was transmitted to MMF. The design of repeated SPR excitations in an Au-coated SMF improved the sensing efficiency and reduced the sensor size (Fig. 3A). To characterize the ability of our fiber probe to detect surface loading, liquids with different refractive indices were prepared. The sensing spectral peak exhibited a red shift of wavelength (Δλ_I_) in detecting liquid with an increased refractive index from 1.3334 to 1.3642 (Fig. 3B), while a reduction of peak wavelength (Δλ_D_) was observed in detecting liquid with a decreased refractive index from 1.3699 to 1.3400 (Fig. 3C). As a result of the Au-coated surface sensing area on the fiber, variations of liquid refractive index (equivalent to density) could directly mimic the changes in surface loading. To further explore the responsive capability of our sensor, a series of rising and descending refractive indices were detected by the fiber probe. The spectral peak wavelengths were consistent with surface loading for all detection orders (Fig. 3D). The stability of detection at each refractive index step was also calculated based on 20 consecutive measurements of each liquid within 1 min. For the 13 liquid samples, the detection stability was represented as an average standard deviation of only 0.06 nm, indicating the high sensing reliability of the fiber probe (Fig. 3E).

### Fabrication of plasmonic fiber probe

**Fig. 4.**
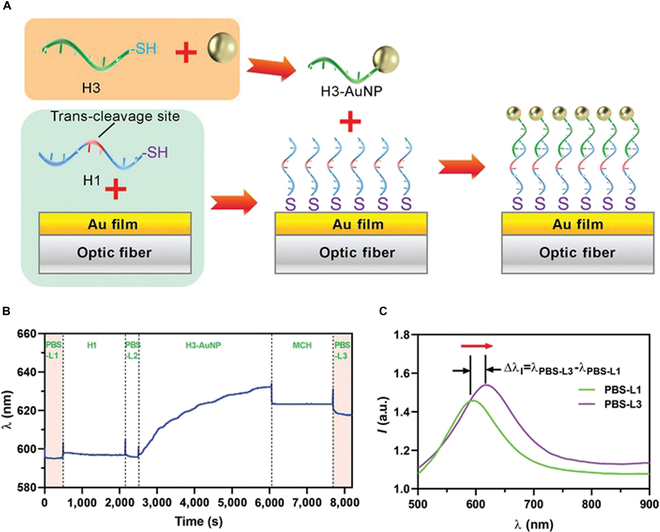
Fabrication of plasmonic fiber probe. (A) Schematic view of fiber probe fabrication procedure. (B) Changes of SPR signals shown as wavelength during fabrication. (C) Comparison of spectra recorded at beginning and end of fabrication process. Δλ_I_ indicates the change of SPR signals at final (λ_PBS-L3_) and initial (λ_PBS-L1_) PBS equilibration stages.

Cas12a-mediated *trans*-cleavage requires ssDNA (H1, 5′-SH-CTTTACTCAAC**TTATTATT**ACGAACATCAGG-3′), which contains a region of TA-rich bases as the cleavage site for Cas12a enzyme. The ultrafine fiber was coated with a gold film for DNA oligo conjugation via Au–S bonding. To facilitate the attachment of gold nanoparticles (AuNPs) onto the fiber surface, an ssDNA oligo (H3) was synthesized with 7 bases at the 5′ end complementary to the 3′ end of H1. The thiolated form of H3 was ligated to 15-nm AuNPs to generate H3-AuNP structures. To prepare the functional sensor, thiolated H1 was first conjugated to the sensor surface via Au–S bonding. Hydrogen bonds then facilitated the continuous binding of H3-AuNP to the fiber-ligated H1, leading to the immobilization of AuNPs on the sensor surface (Fig. 4A).

A micro-spectrometer was used to monitor the SPR spectra in real time. After initial equilibration with phosphate-buffered saline (PBS), the fiber probe was first incubated with H1 DNA. Subsequent incubation with H3-AuNPs caused a rapid increase in the SPR wavelength from approximately 595 to 630 nm within 1 h, suggesting the successful modification of the fiber surface with AuNPs. The fiber coating was washed with 6-mercapto-1-hexanol (MCH) solution to eliminate physically absorbed AuNPs and excessive ssDNA oligos. The SPR wavelength decreased by approximately 8 nm after washing with MCH solution and by 2 nm after PBS equilibration (Fig. 4B). During the sensor preparation process, the spectral peaks of each step were recorded and an overall red shift in the SPR peak was observed after the immobilization of AuNPs on fiber surface (Fig. 4C).

### Preparation of Cas12a-mediated viral DNA sensing system

**Fig. 5.**
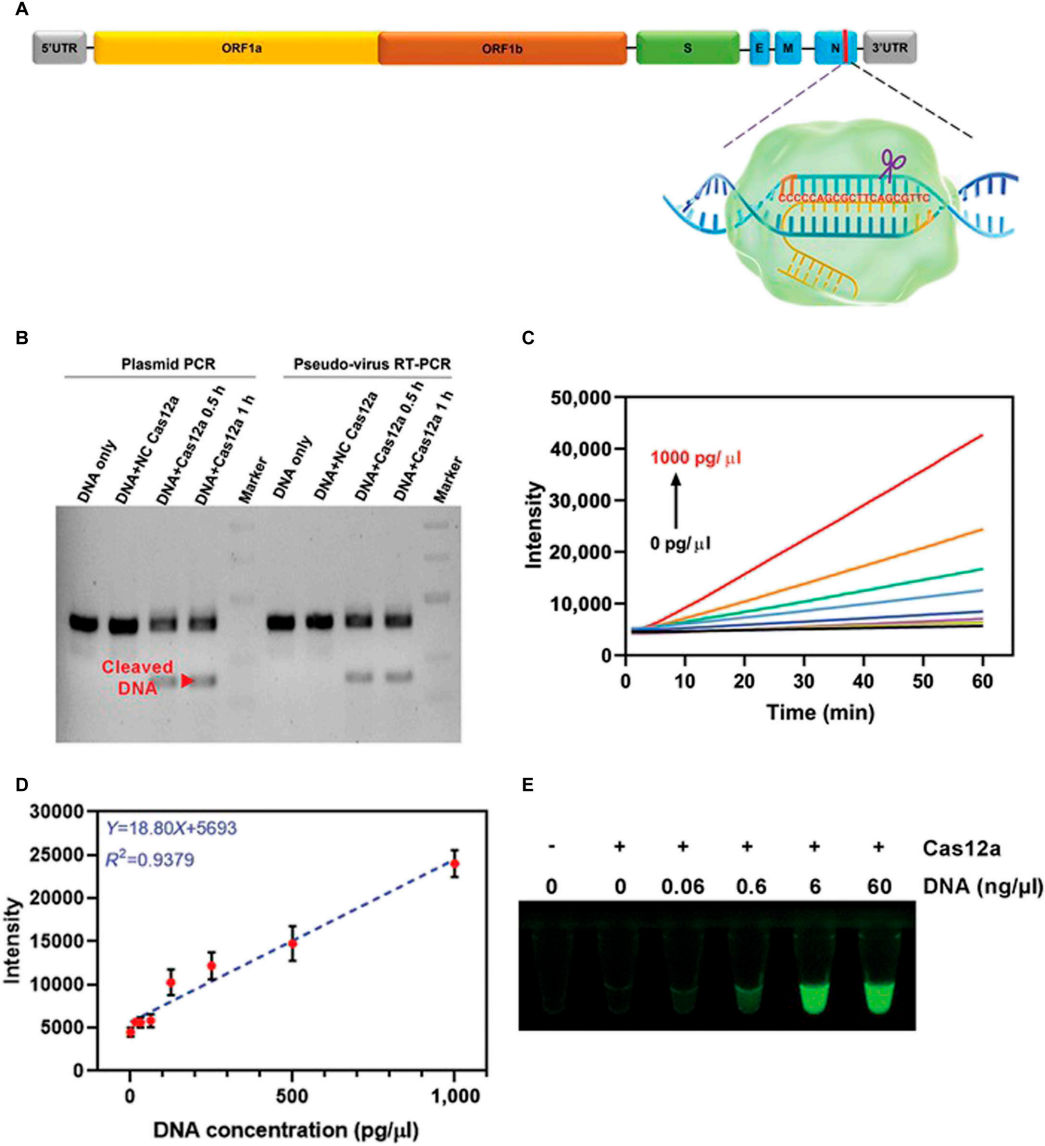
Sensing of N gene of SARS-CoV-2 by Cas12a. (A) Graphical view of Cas12a RNP targeting a consensus sequence within N gene of viral genome. (B) Gel electrophoresis of Cas12a-digested DNA. Red arrow: Cleaved DNA fragment. (C) Fluorescent signals generated by reporter assay from various concentrations of DNA substrate. (D) Linear fitting of fluorescent signals with DNA concentration. Data were shown as mean ± SEM (*n* = 3). (E) Observation of FAM fluorescence produced by Cas12a *trans*-cleavage under UV light.

The SARS-CoV-2 viral genome consists of approximately 30,000 nucleotides, which constitute 12 genes including ORF1a, ORF1b, S, and N. The highly frequent mutations in the S gene have resulted in the emergence of several variants and variant subtypes in the COVID-19 pandemic. By contrast, the N gene coding for the conserved nucleocapsid protein has remained relatively stable during the emergence of the variants. In the present study, a conserved region of approximately 400 nucleotides in the N gene was reverse-transcribed into dsDNA as a Cas12a RNP substrate (Fig. 5A). A pair of specific primers targeting the conserved region of the N gene was designed for qPCR. The targeted DNA fragments were successfully amplified from both the plasmid template and genomic cDNA obtained from the pseudovirus (Fig. S1). The PCR products of the N gene were sequenced using the Sanger method, and the presence of crRNA-targeting bases was confirmed (Fig. S2).

To test the *cis*-cleaving ability, both the plasmid and viral PCR products were treated with N gene-specific Cas12a RNP. Distinctive bands at approximately 200 base pairs appeared after 30 min of incubation, indicating high specificity and efficiency of the Cas12a detection system (Fig. 5B). The *trans*-cleavage behavior of Cas12a was also examined using the fluorescent reporter assay. A fluorescence-quencher ssDNA reporter was synthesized for Cas12a-mediated cutting. Different concentrations of PCR fragments were incubated with Cas12a RNP and the ssDNA reporter to generate fluorescence curves (Fig. 5C). The fluorescence signal showed a strong positive correlation with DNA concentration at 30 min (equation: *Y* = 18.80*X* + 5,693, *R*^2^ = 0.9379; Fig. 5D). Under ultraviolet (UV) light, FAM fluorescence was enhanced with the increased concentration of DNA (Fig. 5E).

### Performance of CRISPR/Cas12a-guided DNA sensing on fiber

**Fig. 6.**
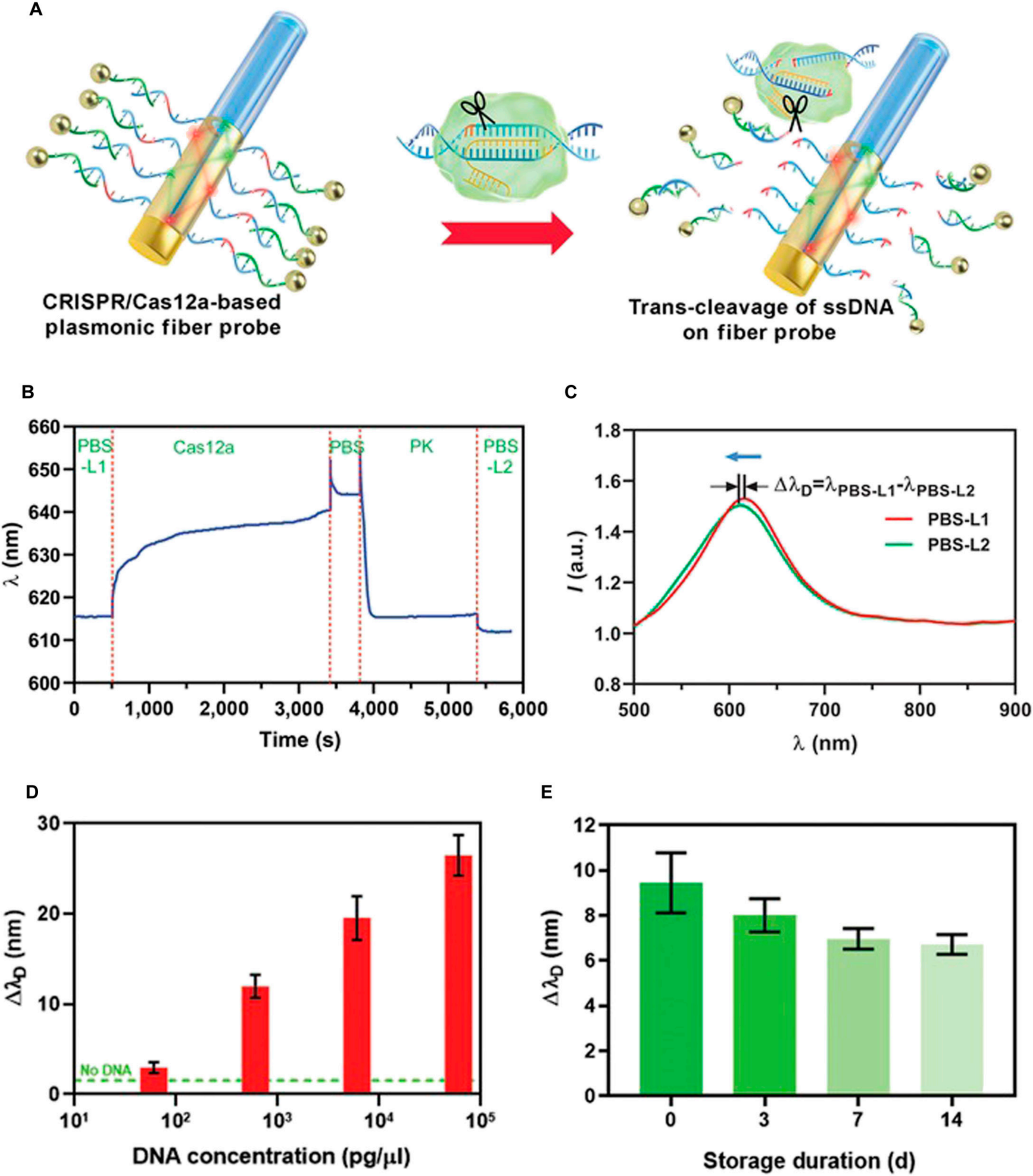
Fiber-optic sensing of viral N gene DNA. (A) Graphical view of Cas12a-mediated cutting of ssDNA immobilized on fiber surface. (B) A typical sensorgram showing Cas12a-mediated DNA sensing and following wash-off by PK. DNA concentration: 60 pg/μl. (C) Spectra shift indicating SPR signals due to Cas12a-mediated release of AuNP. Δλ_D_ reflected the wavelength change between PBS-L1 and PBS-L2 stages. (D) Examples of SPR wavelength changes at different DNA concentrations. (E) Comparison of SPR signals acquired with sensors after different storage periods. Data were shown as mean ± SEM (*n* = 3).

One of the major advantages of fiber sensing is the real-time surveillance of SPR signals. To determine the efficiency of Cas12a-mediated DNA cleavage, a PBS-equilibrated fiber probe with AuNP modification was first incubated with a reaction solution containing Cas12a RNP and N gene DNA for 1 h with rotation to maximize the recognition of the target DNA and the *trans*-cleavage of sensor-ligated ssDNA by Cas12a (Fig. 6A). In a typical test of 60 pg/μl DNA, the adsorption of Cas12a RNP on the fiber probe caused the wavelength to gradually increase from 616 nm (λ_PBS-L1_) to 640 nm. The sensor was then rinsed and subjected to proteinase K (PK) treatment. Because of the degradation of Cas12a protein on the sensor surface, the wavelength quickly decreased by approximately 30 nm. The final PBS wash (λ_PBS-L2_) of the fiber sensor resulted in a further reduction in wavelength because of the removal of absorbed residual protein, revealing the *trans*-cleavage activities of Cas12a (Fig. 6B). The overall change in SPR signals λ_PBS-L1_ − λ_PBS-L2_ (Δλ_D_) was recorded as a blue shift in the spectral peak (Fig. 6C).

The relationship between the quantity of N gene DNA and SPR signal was also assessed by serially diluting the PCR products, and a positive correlation between the amount of DNA and the change in wavelength was observed (Fig. 6D). The logarithm values of DNA concentration showed strong positive linearity with wavelength changes (Δλ_D_), suggesting that our fiber-sensing technique could be utilized for viral DNA quantification (Fig. S3).

The storage conditions of the fiber probes were also investigated. When immersed in PBS and stored at 4 °C, only a weak reduction of SPR signals was exhibited. On days 3, 7, and 14 after fiber sensor engineering, only a slight decrease in Δλ_D_ was recorded after the reaction with input DNA, suggesting that fiber probes can be stored in a refrigerator for at least 2 weeks (Fig. 6E). The high stability and sensitivity of the plasmonic fiber probes are promising attributes for their large-scale application in rapid POCT.

### SPR sensing of virus-spiked water samples

**Fig. 7.**
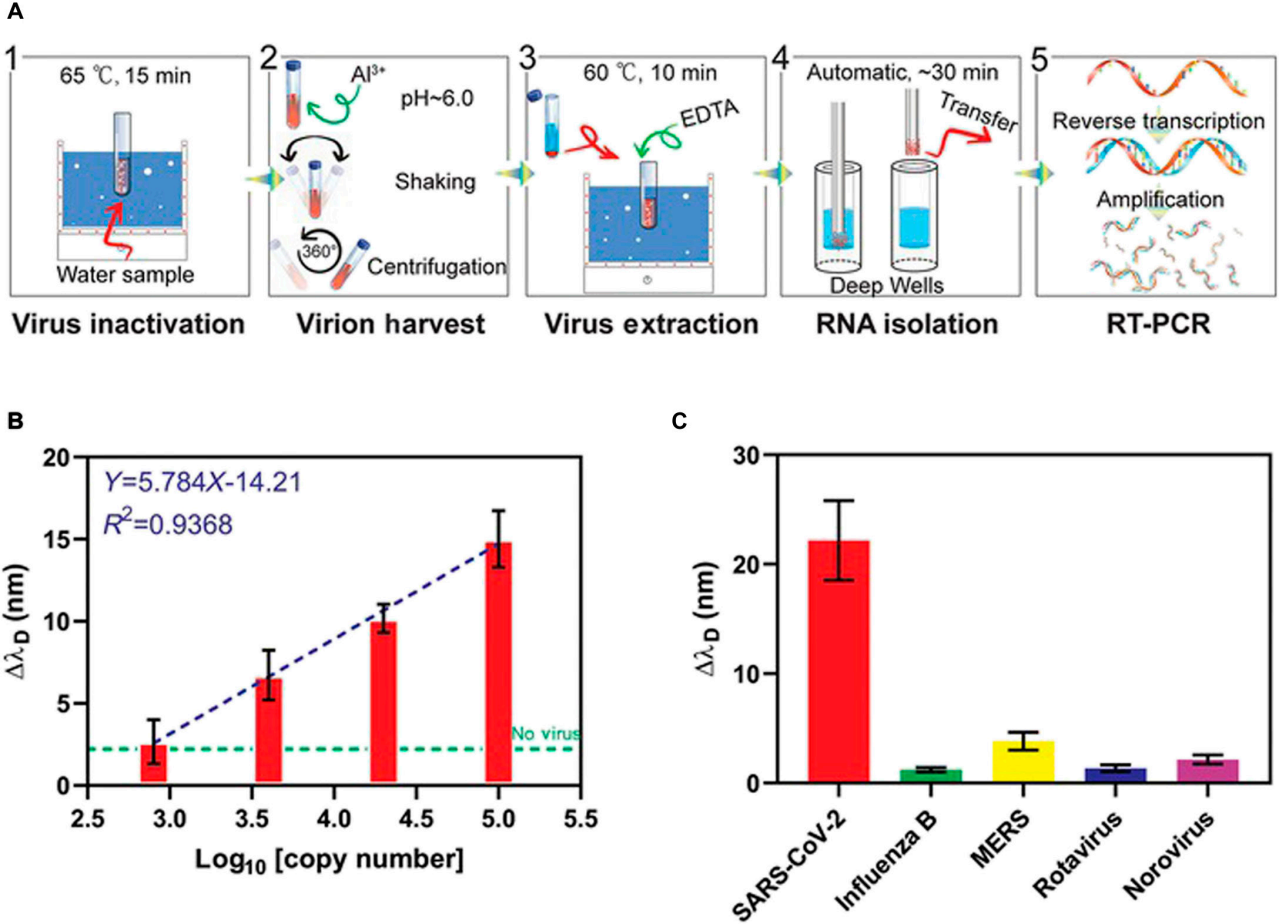
Enrichment and sensing of pseudo-virus spiked into water. (A) Diagram illustrating the virus enrichment procedure. (B) Linear fit between SPR signals and the logarithm of viral copy number. (C) Specificity test of fiber probe for 5 common water-contaminant pathogens. Viral copy number: 5 × 10^6^ copies/ml. Data were shown as mean ± SEM (*n* = 3).

To assess the relationship between viral copy numbers and SPR signals, we spiked the water with SARS-CoV-2 pseudovirus encoding the N gene to mimic pathogen contamination. After initial heat inactivation, virions were precipitated by aluminum hydroxide in weakly acidic conditions and were released when treated with ethylenediaminetetraacetic acid (EDTA). Using an automatic and magnetic RNA extraction apparatus, RNA was harvested and concentrated from the virion solutions within only 30 min. The prepared RNA was reverse-transcribed and amplified using N gene-specific primers (Fig. 7A).

To simulate the natural conditions of viral contamination, pseudoviral particles were spiked into water at concentrations of 800 to 100,000 virions/ml. Considering the limit of detection (LOD) of fluorescence-based CRISPR/Cas12 sensing (~100 copies/ml) [37] and the potential loss of virus during the water processing steps, the cDNA was subjected to 40 cycles of PCR amplification, and around 60 ng/μl N gene-specific DNA was obtained for the sample with the highest viral concentration. The pseudoviral particles were serially diluted and an increase in wavelength was observed, similar to that directly produced by PCR products. SPR signal changes (Δλ_D_) were positively correlated with viral copy number (Fig. S4). The logarithm of the viral copy number showed good linearity with the wavelength change (Δλ_D_), which was consistent with the results of DNA sensing (equation: *Y* = 5.784*X* − 14.21, *R*^2^ = 0.9368; Fig. 7B). The lowest dilution of pseudoviral particles resulted in a Δλ_D_ comparable to that of samples without virions (“No Virus”). The LOD of the plasmonic biosensor was calculated to be ~2,300 copies/ml based on the “3 times standard deviation” principle. The discrepancy between the calculated LOD and the lowest concentration of detectable sample was mainly due to the large standard deviation value generated from 3 independent tests.

The specificity of the fiber probe was evaluated based on its ability to distinguish SARS-CoV-2 from MERS (a coronavirus that shares identical genomic components) [38], influenza B (a common respiratory virus during winter), rotavirus, and norovirus (common causes of diarrhea) [39]. The fiber probe discriminated the COVID-19 genome from the 4 other viruses even at a high copy number of ~5 × 10^6^ copies/ml (Fig. 7C), which also supported its specificity and applicability in sensing SARS-CoV-2 in water samples.

### Sensing of SARS-CoV-2 and its Omicron variant in sewage water

**Fig. 8.**
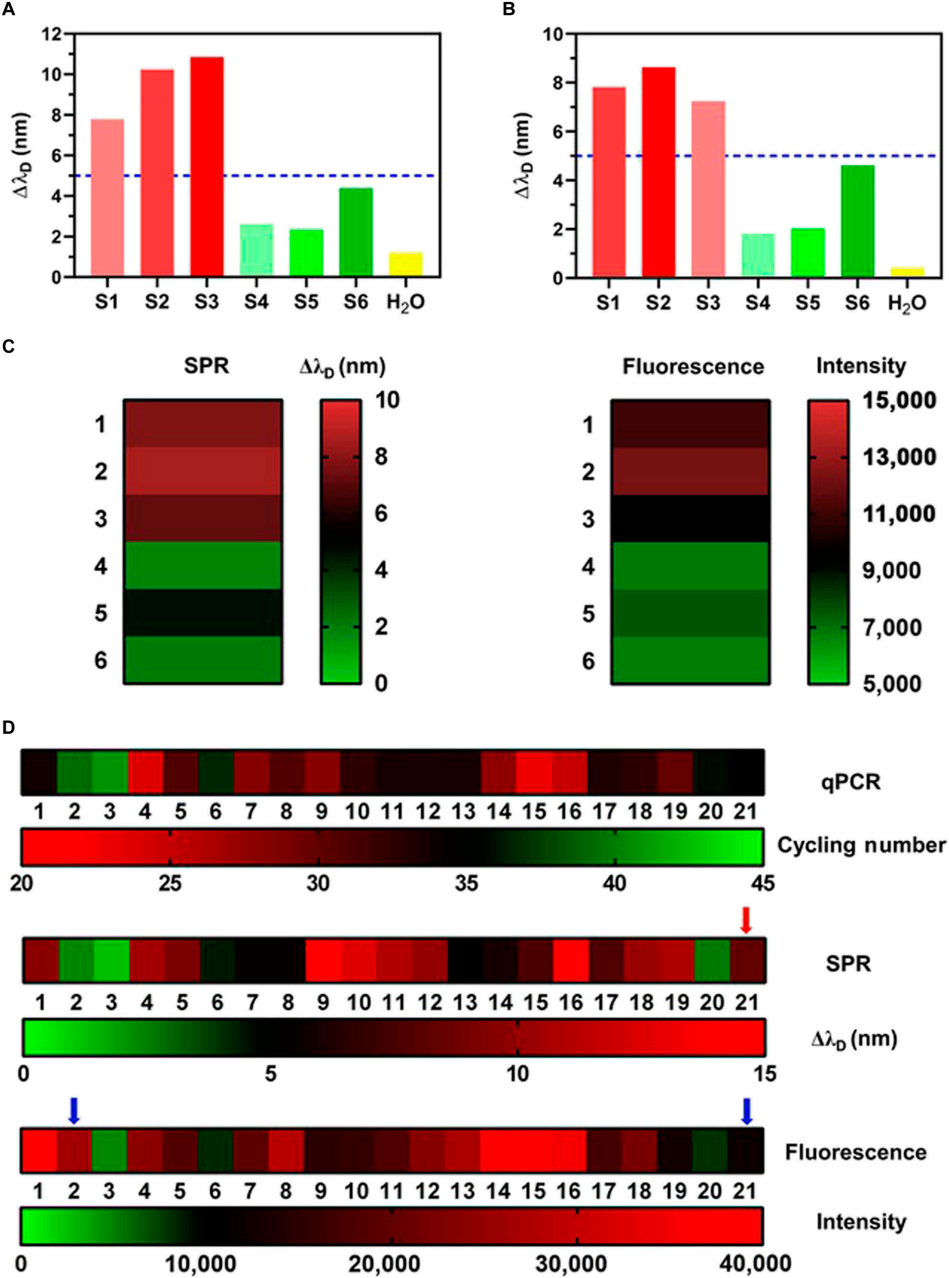
Detection of viral particles in sewage water. (A) SPR signals of N gene for 6 sewage water samples. (B) SPR signals detected due to Omicron L981F mutation. Dashed lines at 5 nm to distinguish samples with high or low level of viral contamination. (C) Heatmaps of SPR and fluorescent readouts from detection of Omicron VOC in sewage water. (D) Summary of viral loading in sewage water samples detected by 3 methods. Red arrow: One readout by SPR measurement inconsistent with qPCR result. Blue arrows: Two readouts by fluorescent reporter assay inconsistent with qPCR result.

Motivated by the above-mentioned results, we also tested viral contamination in urban sewage water. For qPCR, the diagnostic Ct for clinical throat swabs is 30 to 35 [40]; however, there is no definite Ct cutoff for water samples. Initially, the focus was on a group of 6 samples with varying Ct values determined by qPCR (Fig. S5). The viral load of the water samples was also examined using fluorescence-based approach. A steady increase in fluorescence intensity was detected, which exceeded 19,900 at 30 min for all 3 positive samples (Fig. S6). The corresponding SPR signals were also monitored and all 3 samples (S1 to S3) exhibited a >5-nm increase in signal strength, while the signal changes for the other samples (S4 to S6) remained below 5 nm (Fig. 8A). The viral copy numbers of the 6 sewage water samples were calculated based on the linear fit equation in Fig. S4. Samples S4 to S6 were estimated to host less than 1,800 copies/ml viral genome, whereas samples S1 to S3 contained more than 5,100 copies/ml (Table S2).

Apart from the assessment of viral load, the determination of virus subtype from sewage water is crucial for the effective management of disease spread and public health. Since the end of 2021, the Omicron variant and its subtypes have rapidly spread across the globe owing to their high transmissibility and infectivity [41]. One of the characteristic mutations, L981F, served as the CRISPR/Cas12a target in the identification of Omicron (Fig. S7). A DNA fragment containing the mutation site was amplified by PCR from plasmids encoding the S gene of either the wild type or Omicron variant (Fig. S8). Cas12a RNP performed *cis*-cleavage of the S gene DNA (Fig. S9), and the presence of a crRNA-targeted sequence was confirmed by sequencing (Fig. S10). The *trans*-cleavage of the fluorescent reporters resulted in the release of FAM signals. The fluorescence intensities from 3 positive samples (S1 to S3) all exceeded 9,000 (Fig. S11). Using plasmonic fiber sensing, more than 5-nm signal changes in samples S1 to S3 were observed (Fig. 8B), which exhibited a similar heatmap pattern when compared to the fluorescent method (Fig. 8C).

To conduct a more comprehensive examination of sewage water samples, we collected a total of 21 tubes of sewage water from variable sources, such as hospitals, natural water reserves, and urban households (Table S3). To ensure the diversity among the samples, the collection sites were dispersed across several cities in South China. Despite the lack of a well-recognized Ct threshold to discriminate out virus-contaminated environmental samples, relatively low Ct values generated by qPCR indicated relatively high risk of COVID-19 transmission through water source. Among the samples in this study, 16 were considered as highly “virus-contaminated” based on their relatively low Ct values (Ct < 35 in this study). Considering the 5-nm threshold for SPR measurement and the intensity cutoff of 15,000 for fluorescent reporter assay, both methods successfully detected these 16 samples. For the remaining 5 samples, one SPR result (red arrow) and 2 fluorescent reporter readouts (blue arrows) were inconsistent with their respective qPCR results. For the outcomes of these 3 techniques, the generated heatmaps presented a similar color pattern (Fig. 8D). The consistency of measurement results among the 3 methods supported the applicability of our portable sensor system for sensing COVID-19 in water.

## Discussion

The precise and efficient detection and surveillance of pathogenic microorganisms in environmental samples are urgently needed for the early prevention of infectious epidemic. In the present study, a plasmonic fiber biosensor has been fabricated by immobilizing AuNPs on the fiber surface, providing fast and accurate sensing of pathogen genomes. Upon exposure to viral targets, the *trans*-cleavage of ssDNA and the release of AuNPs by the Cas12a complex from the fiber surface collaboratively cause a shift of the spectral peak that is proportional to the viral copy number. The LOD of our fiber-optic SPR biosensor is approximately 2,300 viral copies/ml, which is functionally comparable to fluorescent reporter assays (around 100 to 10,000 copies/ml). Regarding SARS-CoV-2 in sewage water samples, the detection results generated by plasmonic fiber probe are consistent with those of qPCR and fluorescence reporter assay. The virus sensing and genotyping procedure can be completed in less than 2 hours without loss of accuracy.

Despite the end to COVID-19 as a worldwide health emergency declared by WHO, the outbreak of other infectious diseases still poses potential threats to public safety. The surveillance of pathogens by the biosensor mainly relies on the unique recognition of microorganism DNA/RNA by CRISPR enzyme. Because of the facile design of highly specific crRNAs based on bioinformatics, a series of Cas12a RNPs can be assembled to collaborate with the biosensor for the fast and accurate sensing of pathogens. Considering the recently reported cases of monkey pox virus transmission worldwide, a Cas12a complex targeting viral F8L gene has been prepared and the biosensor has successfully detected fewer than 60 copies/μl DNA spiked in blood samples [42], which further support the applicability of our device in the surveillance of other infectious diseases.

Owing to its small scale and ease of assembly, the portable fiber biosensor with microorganism enrichment devices can be installed adjacent to water sources or urban drainage pipelines to function as a next-generation device for the POCT of pathogen contamination. Our miniaturized biosensor only consists of 4 main components, i.e., a light source, a spectrometer, a thermostat, and a removable fiber tip, occupying a limited space of around 0.02 m^3^. At the proximity of water sources, environmental samples are collected to harvest nucleic acids at high purity with an automatic and magnetic DNA/RNA extraction apparatus. If necessary, the obtained DNA can be amplified isothermally or by PCR. When DNA samples are subjected to the incubation with Cas12a and fiber tips, indications of microorganisms like copy number or mutation can be promptly reported by the biosensor in situ. The pathogens in water can be monitored in real time, and the performance of CRISPR/Cas-assisted genotyping provides useful information for predicting disease transmission patterns. This study offers a rapid, sensitive, and accurate solution for early monitoring the transmission and the evolution of infectious diseases.

## Materials and Methods

### Reagents

SMFs with 9-μm core and MMFs with 50-μm core were obtained from Yangtze (China). Fiber cutters and fusion splicer electrodes were purchased from Fujikura (Japan). An Au target with 99.99% purity was purchased from ZhongNuo Advanced Material (China). Tin foils were acquired from Rigorous (China). Aluminum chloride (AlCl_3_; A822326), EDTA (E809068), tris(2-carboxyethyl)phosphine hydrochloride (TCEP; T819166), and MCH (M814343) were acquired from Maclin Chemicals (China). AuNP solution (10 nm) was purchased from Nanoeast (China). Cas12a enzyme was obtained from EZ Assay Biotech (China). Agarose (G5056), PBS (G4202), and tris–acetate–EDTA buffer (TAE; G3001) were purchased from Servicebio (China). DNA oligos were synthesized by Azenta (China). Custom-made crRNAs were provided by Sangon (China). Sequence information of DNA oligos and crRNA was provided in Table S1. SARS-CoV-2 pseudo-viruses (1 × 10^8^ copies/ml) packaging N gene and S gene were purchased from Zoonbio (China). Viral genomic fragments of Influenza B, MERS, rotavirus, and norovirus were stored in our laboratory. DNase- and RNase-free water was purchased from Tiangen (China). All chemicals were of analytical-grade purity and directly used without purification.

### Fabrication of CRISPR-based plasmonic fiber probe

A plug-in plasmonic fiber probe was designed for viral sensing in collaboration with CRISPR/Cas12a system. An MMF was first cut by a fiber cutter to obtain a flat end. Similarly, 2 flat ends were obtained under the same operation for an SMF with the length restricted to 5 mm. The flat end faces of MMF and SMF were aligned and spliced by fusion splicer electrodes to form an MMF-SMF hybrid structure. Next, the SMF portion was wrapped with tin foils to avoid contamination by end-face coating, and a 250-nm Au film was deposited on the exit end of SMF. After removing the tin foils, the cylindrical surface of the SMF part was finally decorated with 50-nm Au film by a rotator [43].

Surface immobilization of AuNPs on optic fiber probe was conducted as previously described [44]. Briefly, DNA oligos (H1 and H3, 100 μM) with 5′ disulfide bond modification were treated with TCEP solution (10 mM) for 1 h to expose the free -SH group. H1 DNA (20 μl) was diluted with PBS for downstream applications. H3 DNA (20 μl) was mixed with AuNP solution (200 μl) and kept at −80 °C refrigerator overnight. The following day, the mixture was thawed to obtain H3-modified AuNPs (H3-AuNPs). The AuNPs with ssDNA conjugation were observed under a Hitachi HT7700 (Japan) transmission electron microscope (Fig. S12).

To prepare the AuNP-fabricated biosensor, as-prepared plasmonic fiber probes were initially immersed in PBS for 5 min. The fiber probes were then incubated in a reservoir of H1 solution for 30 min with gentle rotation. After a brief wash of PBS (5 min), fiber probes were moved into H3-AuNP solution and incubated for 1 h under stirring. Finally, they were treated with MCH solution (1 mM, 30 min) and washed with PBS for virus detection. Fiber probes with AuNP immobilization can be maintained at 4 °C for long-time storage.

### Fiber-optic SPR device

The SPR device is composed of a light source (HL2000-12, Fu Xiang, China), a spectrometer with a spectral range of 400 to 1,000 nm (USB 4000, Ocean Optics), a Y-splitter coupler, and a thermostat (Joanlab, China).

### RT-PCR of viral N gene

Plasmid templates containing the N gene or S gene of SARS-CoV-2 were obtained from Azenta (China). To amplify N gene-specific sequence from pseudo-virus-spiked or environmental water samples, 10 μl of extracted viral RNA was reverse-transcribed into cDNA using SweScript All-in-One First-Strand cDNA Synthesis Super Mix for qPCR (G3337, Servicebio) according to the manufacturer’s protocol.

In a typical 20-μl reaction system, the DNA fragment copies were amplified from the above DNA template (plasmid: 100 pg, cDNA: 1 μl) after 40 cycles of PCR program (95 °C for 15 s, 57 °C for 15 s, and 72 °C for 30 s) using Taq DNA polymerase (G3441, Servicebio). The PCR products were analyzed with agarose gel and sequenced by the Sanger method.

### Fluorescent reporter assay

FAM fluorescent intensity was measured in the Cas12a reporter assay to quantify the N gene DNA. PCR-amplified DNA (1 μl) was diluted into 20 μl Cas12a digestion system consisting of 1 pmol of Cas12a enzyme, 1 pmol of crRNA, and 100 pmol of ssDNA reporter. The emission of FAM fluorescence was monitored on the 7500 Real-Time PCR System (Applied Biosystems) at 1-min intervals.

### Cas12a RNP-mediated cleavage of ssDNA on fiber

The as-prepared fiber probes were first equilibrated with PBS to record the baseline SPR signals (λ_PBS-L1_). Sensors were then dipped into the reaction reservoir (300 μl) containing Cas12a enzyme (20 pmol), crRNA (20 pmol), and 1 μl RT-PCR product with gentle shaking at 37 °C for 1 h. The fiber probes were briefly washed with PBS and subjected to treatment with PK (100 ng/ml, 37 °C, 30 min). Finally, fiber probes were inserted into the PBS solution again to acquire the cleavage signal (λ_PBS-L2_). The wavelength shift (Δλ_D_, λ_PBS-L1_ − λ_PBS-L2_) served as an indicator of viral DNA concentration.

### RNA isolation from water samples

Virus enrichment and RNA extraction were conducted in the class II B2 biosafety cabinet following the method for enrichment and nucleic acid detection of SARS-CoV-2 in sewage water (WS/T 799-2022, China).

Virus-spiked water or sewage water samples collected from different sources (40 ml) were first heated at 65 °C for 30 min to inactivate viruses. After centrifugation at 1,000 rpm, the supernatant was collected and aluminum chloride solution (AlCl_3_; 300 mM) was added until pH reached 6, which was confirmed by test strips (Shanghai SSS Chemicals, China). Samples were then shaken for 15 min and centrifuged at 8,000 rpm for 5 min to precipitate virus-absorbed aluminum hydroxide colloid. The precipitants were harvested and mixed with 2% EDTA solution at 60 °C for 10 min to release viral particles. Viral RNA was extracted on the MS-002 automatic purification system (Metasensing, China) using an RNA extraction and purification kit (Da’an, China) per the manufacturer’s instructions. Isolated RNA was dissolved into 20 μl of water for downstream experiments.

### Statistics

Output spectra were normalized by SPR Online V2.1 software coded with JAVA. Data are presented as mean ± SEM (*n* = 3). Data analysis was conducted on GraphPad Prism 8 software.

## Data availability

All data relevant to this study are available in the manuscript and supporting information. Data related to sewage water samples are provided in Tables S2 and S3. Raw data are available from the corresponding authors upon reasonable request.
