# Supplementary material for: Early and Sensitive Detection of Pathogens for Public Health and Biosafety: An Example of Surveillance and Genotyping of SARS-CoV-2 in Sewage Water by Cas12a-Facilitated Portable Plasmonic Biosensor
Source: Research (Wash D C). 2023 Jul 28;6:0205. doi: 10.34133/research.0205 (PMC10380551; doi:10.34133/research.0205)
Supplement: Supplementary 1 — Figs. S1 to S12 Tables S1 to S3 [file research.0205.f1.docx]

Supplemental Materials

Early and Sensitive Detection of Pathogens for Public Health and Biosafety: An Example of Surveillance and Genotyping of SARS-CoV-2 in Sewage Water by Cas12a-facilitated Portable Plasmonic Biosensor

Short title: COVID-19 Detection and Genotyping by CRISPR Plasmonic Sensor

Tianzhong Li^#^, Yuzhi Chen^#^, Zhi Chen, Yuan Hao, Minyi Liang, Yingxia Liu, Guanyong Ou, Huanian Zhang, Yuxuan Tang, Yabing Hao, Swelm Wageh, Omar A. Al-Hartomy, Abul Kalam, Bin Zhang, Xin Shi, Xuejin Li^†^, Han Zhang^†^

1.


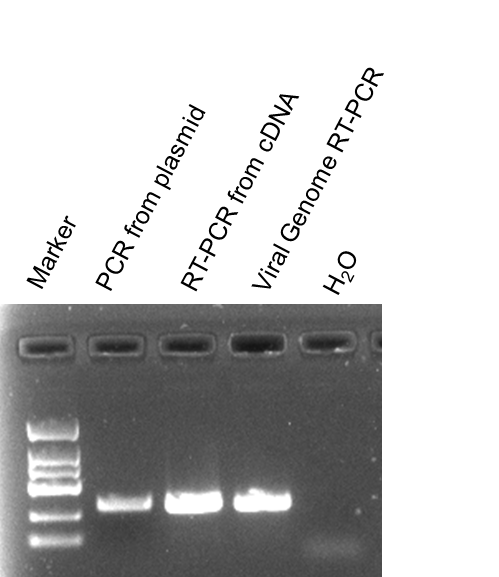


Figure S1. Gel electrophoresis of PCR-amplified DNA of N gene.

2.


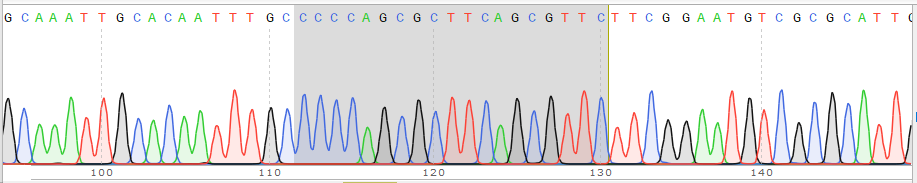


Figure S2. Sequencing result of N gene DNA. The targeted bases were marked as grey.

3.

Figure S3. Linear fit between SPR signals and the logarithm of input DNA concentration (pg/μL).

4.

Figure S4. Relationship between SPR signals and viral copy numbers.

5.

Figure S5. Fluorescent growth curves recorded during qPCR detection of viral N gene.

6.

Figure S6. FAM signals of N gene emitted from six water samples subjected to fluorescent reporter assay.

7.


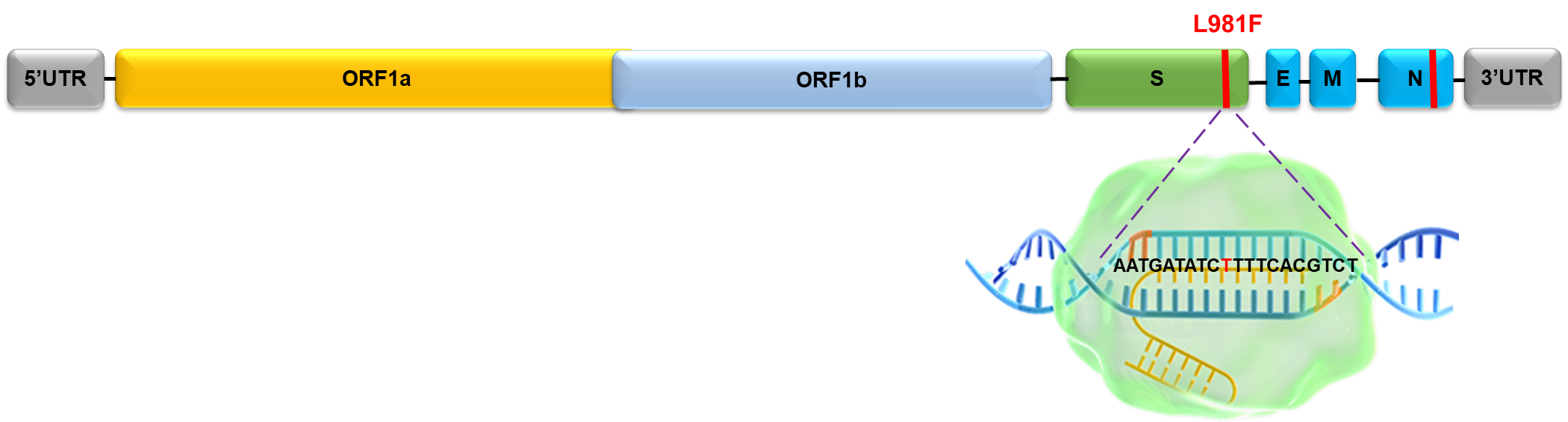


Figure S7. Location of L981F mutation in viral genome and the targeting sequence of Omicron-specific Cas12a enzymatic complex.

8.


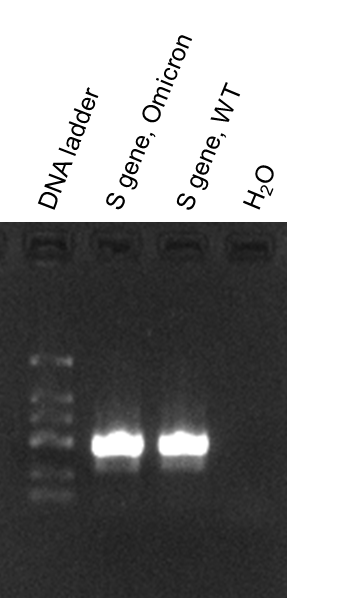


Figure S8. Gel electrophoresis of DNA amplified from plasmids encoding either Omicron or wild type S gene.

9.


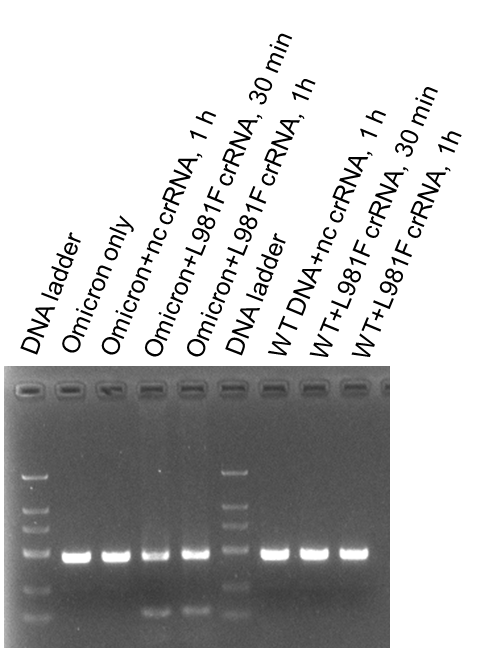


Figure S9. Cleavage of S gene DNA by Cas12a RNP visualized by electrophoresis. Red arrow: cleaved bands.

10.


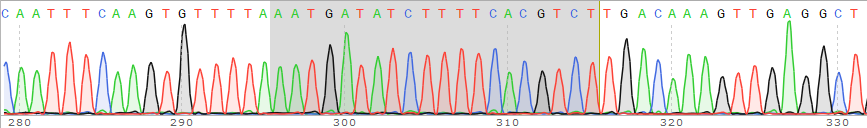


Figure S10. DNA sequencing result of S gene with Omicron L981F mutation. The target of Cas12a crRNA was labeled in grey.

11.

Figure S11. Detection of L981F mutation of S gene in sewage water by Cas12a-based fluorescent assay.

12.


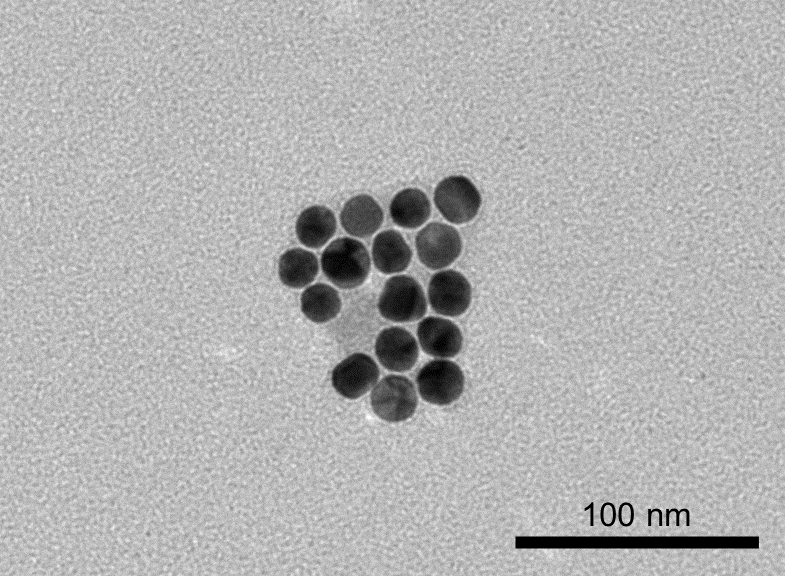

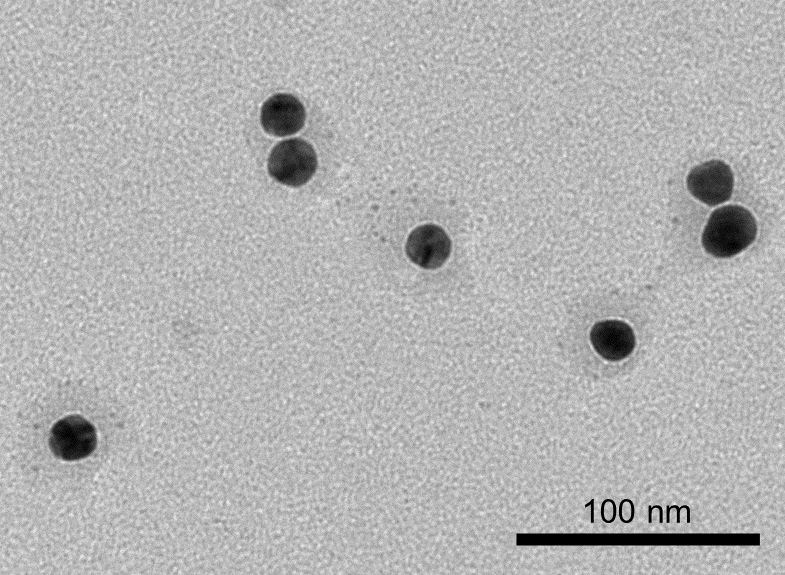
A B


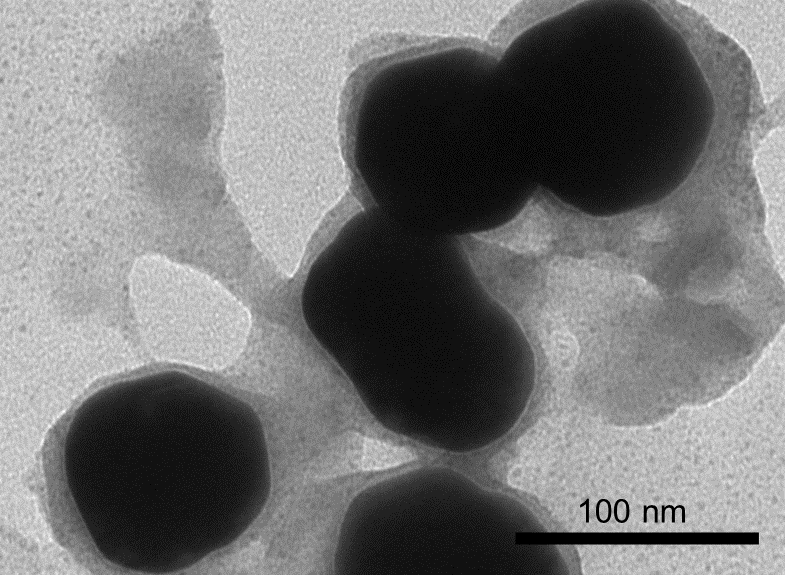

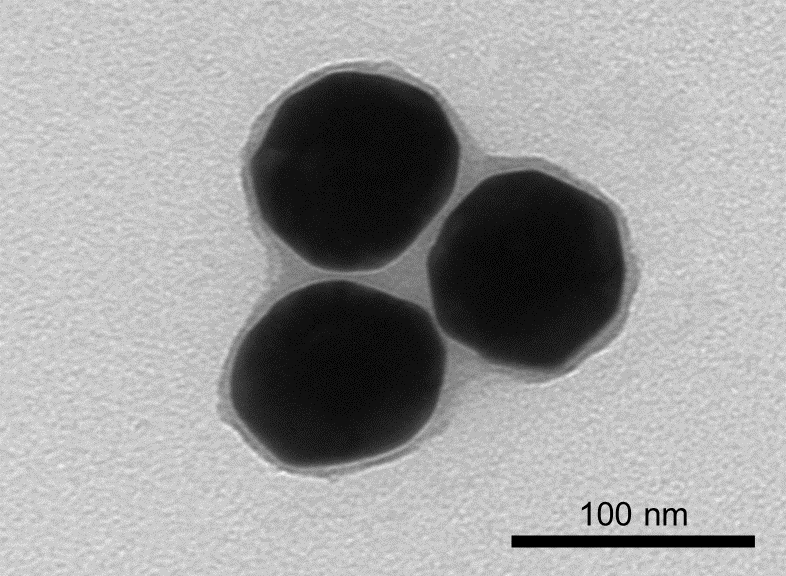
C D

Figure S12. Transmission electron microscopic images of gold nanoparticles (AuNPs). (A) 15-nm AuNPs. (B) 15-nm AuNPs with surface modification of H3 DNA. (C) 60-nm AuNPs. (D) 60-nm AuNPs with surface modification of H3 DNA. Scale bar: 100 nm.

Table S1.

| Name | Sequence (5’-3’) |
| --- | --- |
| N_primer_F | AAACTGTCACTAAGAAATCTGC |
| N_primer_R | GAGTTTCATCAGCCTTCTTC |
| S_primer_F | GCTCAATACACTTCTGCACTG |
| S_primer_R | AGCAAGATTAGCAGAAGCTC |
| N_crRNA | UAAUUUCUACUAAGUGUAGAUCCCCCAGCGCUUCAGCGUUC |
| L981F_crRNA | UAAUUUCUACUAAGUGUAGAUAATGATATCTTTTCACGTCT |
| ssDNA_reporter | FAM-TTATT-BHQ |
| H1 | SH-CTTTACTCAACttattattACGAACATCAGG |
| H3 | SH-CCTGATGTTCGT |

Table S1. Primers, crRNAs, reporters, and DNA oligos used in this study.

Table S2.

| **No.** | **Name** | **qPCR**  **(Ct)** | **Fluorescent Reporter**  **(N Gene)** | **Δλ_D_ of N (nm)** | **Fluorescent Reporter**  **(L981F)** | **Δλ_D_ of L981F (nm)** | **Copy Number (🞩10^3^/mL)** |
| --- | --- | --- | --- | --- | --- | --- | --- |
| 1 | S1 | 33.02 | 25508 | 7.78 | 11019 | 7.82 | ~6.44 |
| 2 | S2 | 32.32 | 31683 | 10.23 | 12274 | 8.64 | ~8.93 |
| 3 | S3 | 29.79 | 19905 | 10.85 | 9374 | 7.24 | ~5.12 |
| 4 | S4 | 35.78 | 14001 | 2.62 | 6777 | 1.81 | ~0.59 |
| 5 | S5 | 39.24 | 36990 | 2.34 | 7517 | 2.02 | ~0.63 |
| 6 | S6 | 36.71 | 14243 | 4.39 | 6697 | 4.62 | ~1.80 |
| 7 | H_2_O | N.A. | 4287 | 1.19 | 5507 | 0.40 | N.A. |

Table S2. Statistical comparison of three detection methods for SARS-CoV-2 in 6 sewage water samples.

Table S3.

| **Sample No.** | **Source** | **qPCR**  **(Ct)** | **Fluorescent Reporter**  **(N Gene)** | **Δλ_D_ of N**  **(nm)** |
| --- | --- | --- | --- | --- |
| 1 | Lake | 33.63 | 46251 | 9.63 |
| 2 | Household | 39.24 | 36990 | 2.34 |
| 3 | Household | 40.86 | 10366 | 1.41 |
| 4 | Hospital | 23.63 | 34357 | 10.50 |
| 5 | Hospital | 30.61 | 27530 | 9.19 |
| 6 | Lake | 36.71 | 14243 | 4.39 |
| 7 | Hospital | 28.14 | 28314 | 5.45 |
| 8 | Hospital | 30.61 | 22673 | 5.50 |
| 9 | Hospital | 28.05 | 39609 | 14.21 |
| 10 | Hospital | 32.32 | 23597 | 12.62 |
| 11 | Hospital | 33.38 | 26728 | 11.01 |
| 12 | Hospital | 33.28 | 33505 | 9.99 |
| 13 | Hospital | 33.40 | 38014 | 5.20 |
| 14 | Hospital | 27.63 | 46532 | 6.04 |
| 15 | Hospital | 22.59 | 42192 | 7.64 |
| 16 | Hospital | 24.78 | 47294 | 14.82 |
| 17 | Hospital | 33.02 | 25508 | 7.78 |
| 18 | Hospital | 32.32 | 31683 | 10.23 |
| 19 | Hospital | 29.79 | 19905 | 10.85 |
| 20 | Household | 35.78 | 14011 | 2.62 |
| 21 | Household | 35.12 | 19188 | 8.42 |

Table S3. Experimental statistics of virions in sewage water samples acquired by three methods. A conserved sequence in viral N gene served as the target.
